# Adenosine A2A Receptor: A New Neuroprotective Target in Light-Induced Retinal Degeneration

**DOI:** 10.3389/fphar.2022.840134

**Published:** 2022-03-21

**Authors:** Manuel Soliño, Ignacio M. Larrayoz, Ester María López, Manuel Rey-Funes, Mariana Bareiro, Cesar Fabián Loidl, Elena Girardi, Alfredo Martínez, Juan José López-Costa

**Affiliations:** ^1^ Instituto de Biología Celular y Neurociencia “Prof. E. De Robertis” (IBCN), UBA-CONICET, Facultad de Medicina, Universidad de Buenos Aires, Ciudad Autónoma de Buenos Aires, Argentina; ^2^ Biomarkers and Molecular Signaling Group, Center for Biomedical Research of La Rioja (CIBIR), Logroño, Spain; ^3^ Angiogenesis Study Group, Center for Biomedical Research of La Rioja (CIBIR), Logroño, Spain

**Keywords:** Adenosine, retina, degeneration, A2A receptor, CGS 21680, SCH58261

## Abstract

Continuous illumination induces the degeneration of photoreceptors. This animal model of light-induced retinal degeneration resembles many characteristics of human degenerative diseases of the outer retina, such as age-related macular degeneration. This work aimed to evaluate the potential neuroprotective effect of the modulation of adenosine A2A receptor in the model of light-induced retinal degeneration. Sprague-Dawley rats were intravitreally injected in the right eye with either CGS 21680, an adenosine A2A receptor agonist, or SCH 58261, an adenosine A2A receptor antagonist. Contralateral eyes were injected with respective vehicles as control. Then, rats were subjected to continuous illumination (12,000 lux) for 24 h. Retinas were processed by glial fibrillary acidic protein (GFAP) immunohistochemistry, terminal deoxynucleotidyl transferase dUTP nick end labeling (TUNEL) technique, Western blotting (WB), and quantitative reverse transcription-polymerase chain reaction (qRT-PCR). Another group of rats was subjected to functional studies by electroretinography. Animals treated with CGS21680 showed a significant increase of apoptotic nuclei in the outer nuclear layer and a significant increase of GFAP immunoreactive area of the retinas but did not alter WB nor electroretinography results. qRT-PCR showed that CGS 21680 significantly increased the expression of interleukin-1β. On the opposite, SCH 58261 significantly decreased apoptotic nuclei in the outer nuclear layer and GFAP immunoreactive area of the retinas. It also significantly decreased GFAP and activated caspase-3 levels as measured by WB and preserved retinal function, as treated eyes showed significantly greater amplitudes of a- and b-waves and oscillatory potentials. qRT-PCR revealed that SCH 58261 significantly decreased the expression of tumor necrosis factor-α. These results show that the blockade of the A2A receptor before the start of the pathogenic process is neuroprotective, as it prevents light-induced retinal damage. The use of A2A receptor antagonists deserves to be evaluated in retinal degenerative diseases.

## Introduction

In recent years, the modulation of adenosine receptors has become a possible neuroprotective strategy to treat a wide range of insults and degenerative diseases of the central nervous system (CNS) (Stone et al., 2009). It has been reported that A1 receptor agonists are neuroprotective in animal models of inflammatory, hypoxic, epileptic, and degenerative diseases of the CNS (Boeck et al., 2005; Rosim et al., 2011; Gori and Girardi, 2013), whereas the use of A2A receptor agonists and/or antagonists has been useful against the neurotoxicity of 6-hydroxydopamine (Nobre et al., 2010), spinal cord injury (Paterniti et al., 2011), convulsions induced by pilocarpine (Rosim et al., 2011), memory dysfunction (Kaster et al., 2015), and degenerative diseases such as Alzheimer disease (Canas et al., 2009) and Parkinson’s disease (Gyoneva et al., 2014; Jenner, 2014). A broader review of the neuroprotective role of A2A receptor modulation may be found in Cunha (2016).

The release of adenosine is an important component of the response to ischemic/hypoxic insults of the retina (Roth et al., 1997; Li et al., 1999), probably through the production of hyperemia that protects neurons from glutamate toxicity (Ostwald et al., 1997). The neuroprotective role of adenosine after ischemic retinal injury could be mediated by A1 (A1R) and/or A2 receptors (Housley et al., 2009).

Adenosine A1 and A2A receptors (A2AR) crosstalk with interleukin (IL)-6 and regulate the production of brain-derived neurotrophic factors (Perígolo-Vicente et al., 2014). The A2AR has been described as a transactivator of many other signaling families, including cannabinoids and neurotrophins (Tebano et al., 2010). The effect of adenosine receptors on other neurotrophins as glial cell line-derived neurotrophic factor and vascular endothelial growth factor seems to be responsible for the neuroprotective role of adenosine (Leibovich et al., 2002; Gomes et al., 2009). In the CNS and in the retina, it has been proposed that A2AR also plays a role in the microglial response to neuronal degenerative diseases (Santiago et al., 2014). The neuroprotective role of adenosine has been reviewed in different models of retinal degeneration, including ischemic retinopathy and diabetes (Dos Santos Rodrigues et al., 2015). Using a model of light-induced retinal degeneration (LIRD), which resembles many of the characteristics of age-related macular degeneration and retinitis pigmentosa (RP), we demonstrated that cyclopentyladenosine, an A1R agonist, protects the photoreceptors from light-induced damage, whereas dipropylcyclopentylxanthine, an A1R antagonist, has the opposite action (Soliño et al., 2018). Besides, the neuroprotective role of A2AR antagonists, such as KW6002 and SCH58261, has been demonstrated against the retinal damage induced by ischemia–reperfusion (Madeira et al., 2016a; Boia et al., 2017). Therefore, the present study aimed to investigate if the modulation of A2AR before the exposure to continuous illumination (CI) is able to prevent retinal damage in the model of LIRD as well.

## Materials and Methods

### Animals

Male Sprague-Dawley albino rats (*n* = 46, body weight 200 g, age 60 days) were used.

### Intravitreal Injections Protocol

Intravitreal injections were performed as previously described (Soliño et al., 2018). Briefly, general anesthesia was performed with ketamine (40 mg/kg; Ketamina 50, Holliday-Scott SA, Argentina) and xylazine (5 mg/kg; Kensol, König SA, Argentina), and in addition, local anesthesia was performed with 2% lidocaine (Lidocaine, Richmond SA, Argentina). Intravitreal injections (5 µl) were performed using a Hamilton syringe (Reno, NV, United States) and a 30-gauge needle. The right eyes received either CGS21680 (Abcam plc, Cambridge, United Kingdom, ab120453), an A2AR agonist, or SCH58261 (Sigma-Aldrich Inc., St. Louis, MO, United States, CAS no. 160098-96-4), an A2AR antagonist. Meanwhile, the left eyes, which were the controls (CTL), received the vehicle. As the volume of vitreous of the rat eye is 13.36 ± 0.64 µl (Dureau et al., 2001), the final vitreal concentrations were 0.9 mM for CGS 21680 and 0.066 mM for SCH 58261 in agreement with previous reports (Ongini, 1998; Font et al., 2008).

### Light-Induced Retinal Degeneration Procedure

One hour after intravitreal injections, rats were continuously illuminated for 24 h at 12,000 lux as previously described (Soliño et al., 2018). Groups of 3–5 rats were simultaneously placed in an open white acrylic box of 60 cm × 60 cm x 60 cm with 12 halogen lamps (12 V, 50 W each) located on top. Lighting level and temperature (21°C) were monitored. Then, the retinas were obtained around 2 p.m. and processed for glial fibrillary acidic protein (GFAP) immunohistochemistry (IHC) (*n* = 4), terminal deoxynucleotidyl transferase dUTP nick end labeling (TUNEL) technique (*n* = 4), Western blotting (WB) (*n* = 5), or quantitative reverse transcription-polymerase chain reaction (qRT-PCR) (*n* = 5). In every case, the numbers indicate the number of rats per drug treatment. A separate group of five rats per drug treatment was used for electroretinography (ERG) studies. After performing a basal ERG, rats were subjected to the intravitreal injections of drugs and exposed to CI. A week later, a second ERG was performed. All animals received food and water *ad libitum*. Animal care was performed in accordance with the Association for Research in Vision and Ophthalmology Statement for the Use of Animals in Ophthalmic and Vision Research. The animal model of continuous illumination and the experimental procedure were approved by the Institutional Committee for the Use and Care of Laboratory Animals of the Facultad de Medicina, Universidad de Buenos Aires (CICUAL, Res (CD) 3130/2017).

### Tissue Processing for Immunohistochemistry and Terminal Deoxynucleotidyl Transferase dUTP Nick End Labeling Assay

Eyes containing the retinas were fixed by immersion in a 4% paraformaldehyde solution for 24 h. After cryoprotection in a 30% sucrose solution, the eyes were embedded in gelatine, blocks were frozen, and sections were obtained using a Lauda Leitz cryostat. Sections (thickness: 20 µm) were mounted on gelatine-coated glass slides and processed by IHC or TUNEL techniques.

### Immunohistochemistry Technique

Endogenous peroxidase activity was inhibited by incubation in methanol containing 3% hydrogen peroxide for 30 min. Overnight incubation with GFAP polyclonal primary antibody (Dako Ink, Cat #Z0334, United States, dilution 1:500) was performed at 4°C. Then sections were incubated with biotinylated goat anti-rabbit antibody (Sigma Chemical Co.,MO; Cat #B8895, dilution 1:500) at room temperature (RT) for 1 h followed by ExtrAvidin-Peroxidase^®^ complex (Sigma Chemical Co., MO., United States; Cat E2886, dilution 1:500) at room temperature (RT) for 1 h as well. Development was performed using the DAB/nickel intensification procedure (Hancock, 1984). Controls were performed by omitting primary antibodies.

### Terminal Deoxynucleotidyl Transferase dUTP Nick End Labeling Assay

Cryostat sections were processed using the ApopTag Peroxidase *In Situ* kit (Chemicon Int, CA, United States, S701), following the instructions described in Soliño et al. (2018). Briefly, sections were incubated with terminal deoxynucleotidyl transferase (Chemicon Int, CA, United States, Cat 90,418) (1 h at 37°C) followed by the anti-digoxigenin conjugate (Chemicon Int, CA, United States, Cat 90,420) (30 min at RT). The reaction was developed using the diaminobenzidine/nickel intensification procedure, and sections were counterstained with eosin.

### Image Analysis of Terminal Deoxynucleotidyl Transferase dUTP Nick End Labeling and Glial Fibrillary Acidic Protein Immunostained Sections

Six retinal sections of both eyes from each experimental group were analyzed (CGS21680, *n* = 4; SCH58261, *n* = 4). Anatomically matched areas of retina among animals were selected, and images were taken using a Zeiss Axiophot microscope attached to a video camera (Olympus Q5) under the same light conditions (Soliño et al., 2018).

The following parameters were measured, blind to treatment, on 8-bit images, using the Fiji software (NIH, Research Services Branch, National Institutes of Mental Health, Bethesda, MD, United States):

*GFAP-positive area*: Images of drug-treated and control retinas were randomly selected. The immunoreactive area of the whole section was thresholded. The region of interest (ROI) was the retinal surface between the two limiting membranes where Müller cells extend their processes. The GFAP-positive area was calculated as the percentage of the ROI immunostained by GFAP.

*TUNEL positive nuclei/1,000 µm* ^*2*^: Images of drug-treated and control retinas were randomly selected and thresholded. As for ROI, frames of 1,000 µm^2^ were randomly determined on the outer nuclear layer (ONL) of treated and control retinas. The analyzed particles function of Fiji was used (Grishagin, 2015), and the TUNEL positive nuclei/1,000 µm^2^ ratio was then obtained for each ROI.

### Western Blotting

The procedure was performed as previously described in Soliño et al. (2018). Retinas were homogenized (1:3, w/v) in lysis buffer (100 mM NaCl, 10 mM Tris-HCl, 0.5% Triton X-100) plus 50 µl of protease inhibitor cocktail (Merck KGaA, Darmstadt, Germany) at 4°C. Protein concentration was determined by the Bradford method. Then, 25 µg of each sample were mixed 4:1 with 5× sample buffer (10% sodium dodecyl sulfate, 0315-M Tris-HCl, 25% beta-mercaptoethanol, 50% glycerol, 0.2-ml bromophenol blue 0.1%, pH 6.8), separated by 15% sodium dodecyl sulfate–polyacrylamide gel electrophoresis and transferred to polyvinylidene difluoride membranes (GE Healthcare Life Sciences, IL, United States). Kaleidoscope Prestained Standards (Bio-Rad Laboratories, CA, United States) were used as molecular weight markers. Membranes were blocked with phosphate-buffered saline/5% nonfat dry milk and incubated overnight at 4°C with either a rabbit polyclonal antibody to GFAP (DAKO Inc., CA, United States, Cat Z0334; dilution 1:500) or a rabbit polyclonal antibody to activated caspase-3 (Sigma Chemical Co., MO., United States; Cat H277. dilution 1:100) and reprobed with a monoclonal anti-β-actin antibody (Sigma Chemical Co., MO, United States, CaT C8487, dilution: 1: 1,000). Membranes were incubated with Amersham ECL donkey anti-rabbit IgG, HRP-linked F (ab)2 fragment, Cat GENA9340, and were developed using a chemiluminescence kit (SuperSignal West Pico Chemiluminescent Substrate, Thermo Scientific, MA, United States, Cat # 34,079). Membranes were exposed to X-ray blue films (Agfa Healthcare, Argentina), which were developed and then scanned with an HP Photosmart scanner. Optical density was quantified using Image Studio Light software. The results were normalized against β-actin.

### Electroretinography

After overnight dark adaptation, rats were anesthesized under dim red illumination with ketamine and xylazine, as was mentioned earlier. An ophthalmic solution containing 5% phenylephrine hydrochloride and 0.5% tropicamide (Fotorretin, Laboratorios Poen, Argentina) was used to dilate the pupils. Recordings were made from both eyes simultaneously (Soliño et al., 2018).

Scotopic ERG: 20 responses to flashes of white light (1 ms, 1 Hz) from a photic stimulator (light-emitting diodes) set at maximum brightness were recorded with an Akonic BIO-PC electroretinograph (Argentina). The registered response was amplified and filtered (1.5-Hz low-pass filter, 500-Hz high-pass filter, notch activated).

Oscillatory potentials (OP): The same photic stimulator was used with filters of high (300 Hz) and low (100 Hz) frequencies. The amplitudes of the OPs were estimated following described methodology (Severns et al., 1994).

The a- and b-waves and OP were measured 40 times, and the values from each eye were averaged. The resultant mean values were used to obtain the group means of a- and b-waves and OP amplitudes ± standard deviation.

### RNA Isolation and Quantitative Reverse Transcription-Polymerase Chain Reaction

The retinas were processed as detailed in Soliño et al. (2018). Briefly, tissues were homogenized with TRIzol (Invitrogen, Madrid, Spain), and RNA was isolated with RNeasy Mini kit (Qiagen, Germantown, MD, United States). Three micrograms of total RNA were treated with 0.5-µl DNAseI (Invitrogen) and reverse-transcribed into first-strand copy DNA using random primers and the SuperScript III kit (Invitrogen). Reverse transcriptase was omitted in control reactions. The resulting copy DNA was mixed with SYBR Green PCR master mix (Invitrogen) for qRT-PCR using 0.3 µM forward and reverse oligonucleotide primers (see Table 1). Quantitative measures were performed using a 7,300 Real-Time PCR System (Applied Biosystems, Carlsbad, CA, USA). Cycling conditions were an initial denaturation at 95°C for 10 min, followed by 40 cycles of 95°C for 15 s and 60°C for 1 min. At the end, a dissociation curve was implemented from 60 to 95°C to validate amplicon specificity. Gene expression was calculated using relative quantification by interpolation into a standard curve. All values were divided by the expression of the housekeeping gene 18S.

**TABLE 1:** List of primers.

Gene	Primer orientation	Primer sequence
TNF-α	Forward	GAG​AGA​TTG​GCT​GCT​GGA​AC
	Reverse	TGG​AGA​CCA​TGA​TGA​CCG​TA
IL-1β	Forward	CCT​CTG​CCA​AGT​CAG​GTC​TC
	Reverse	GAA​TGT​GCC​ACG​GTT​TTC​TT
GFAP	Forward	GAA​GAA​AAC​CGC​ATC​ACC​AT
	Reverse	GGC​ACA​CCT​CAC​ATC​ACA​TC
iNOS	Forward	AGG​CCA​CCT​CGG​ATA​TCT​CT
	Reverse	GCT​TGT​CTC​TGG​GTC​CTC​TG
18 S	Forward	ATG​CTC​TTA​GCT​GAG​TGT​CCC​G
	Reverse	ATT​CCT​AGC​TGC​GGT​ATC​CAG​G

### Statistical Analysis

D’Agostino, KS, Shapiro–Wilk, and F tests confirmed that the data of image analysis of GFAP, IHC, and TUNEL studies of CGS21680-treated rats (*n* = 4) and SCH 58261-treated rats (*n* = 4) display a Gaussian distribution. Then, all the data of this study [GFAP-IHC (*n* = 4), TUNEL (*n* = 4), WB (*n* = 5), qRT-PCR (*n* = 5), and ERG (*n* = 5)] were analyzed using unpaired Student’s *t*-test (GraphPad Software, San Diego, CA, United States). In every case, n is the number of animals per drug treatment. Values are expressed as mean ± standard deviation. Differences were considered significant when *p* < 0.05.

The sample size was calculated based on data published by Soliño et al. (2018). In that study, the number of apoptotic cells, as quantified by TUNEL analysis, was 4.25 positive nuclei/1,000 µm^2^ in animals subjected to LIRD and was reduced to 1.45 when subjects were treated with N6-cyclopentyladenosine, with a standard deviation of 0.74. Free software (http://biomath.info/power/ttest.htm) was used to calculate the sample size. Power was set as 80% for an alpha of 5%, resulting in less than six animals per group to reach a significant improvement of the variable with an unpaired *t*-test.

## Results

### Effects of the Administration of CGS21680 Before Light-Induced Retinal Degeneration

#### Effects on Photoreceptor Apoptosis and Gliosis

**FIGURE 1:**
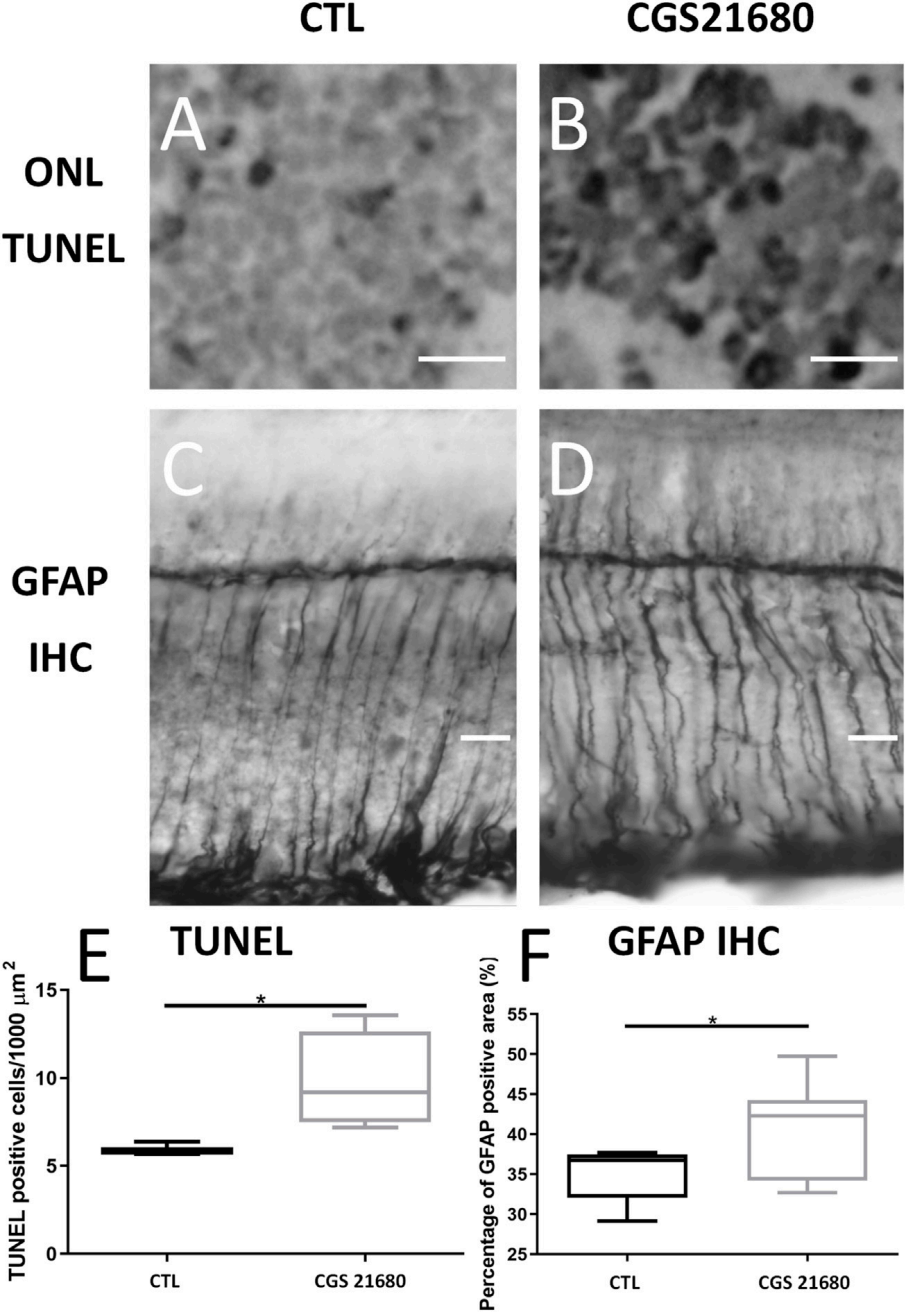
Treatment with CGS21680 increased number of apoptotic nuclei and GFAP immunoreactive areas. **(A,B)** Representative sections showing TUNEL staining of ONL of retina of a CTL eye **(A)** and a CGS21680-treated eye **(B)**. Scale bar: 20 μm. **(C,D)**: GFAP-immunostained sections from retina of a CTL eye **(C)** and a CGS21680-treated eye **(D)**. More intense GFAP immunoreactivity of Müller cells is observed in retina of CGS21680-treated eye compared with CTL. **(E)** Quantification of ONL TUNEL-positive cells. CGS21680 produced a significant increase in positive nuclei of ONL compared with CTL (Student’s *t*-test, *p < 0.05). **(F)**. Quantification of GFAP immunoreactive area. Boxes represent 25 and 75 percentiles, whiskers represent minimum and maximum values, and transverse lines represent medians. CGS21680 produced a significant increase in expression of GFAP compared with CTL (Student’s *t*-test, *p < 0.05).

In the sections stained with the TUNEL technique, a greater density of positive nuclei was observed in the ONL of the retina of CGS21680-treated eyes (9.78 ± 2.752 *vs*. 5.974 ± 0.3612, *p* < 0.05, *n* = 4) (Figures 1A,B,E and Supplementary Figure S3).

The retinas of CGS21680-treated eyes showed an increase GFAP immunoreactivity (40.57 ± 5.948% *vs*. 35.18 ± 3.518%, *p* < 0.05) (Figures 1C,D,F).

#### Apoptosis and Glial Reactivity After Administration of CGS21680

**FIGURE 2:**
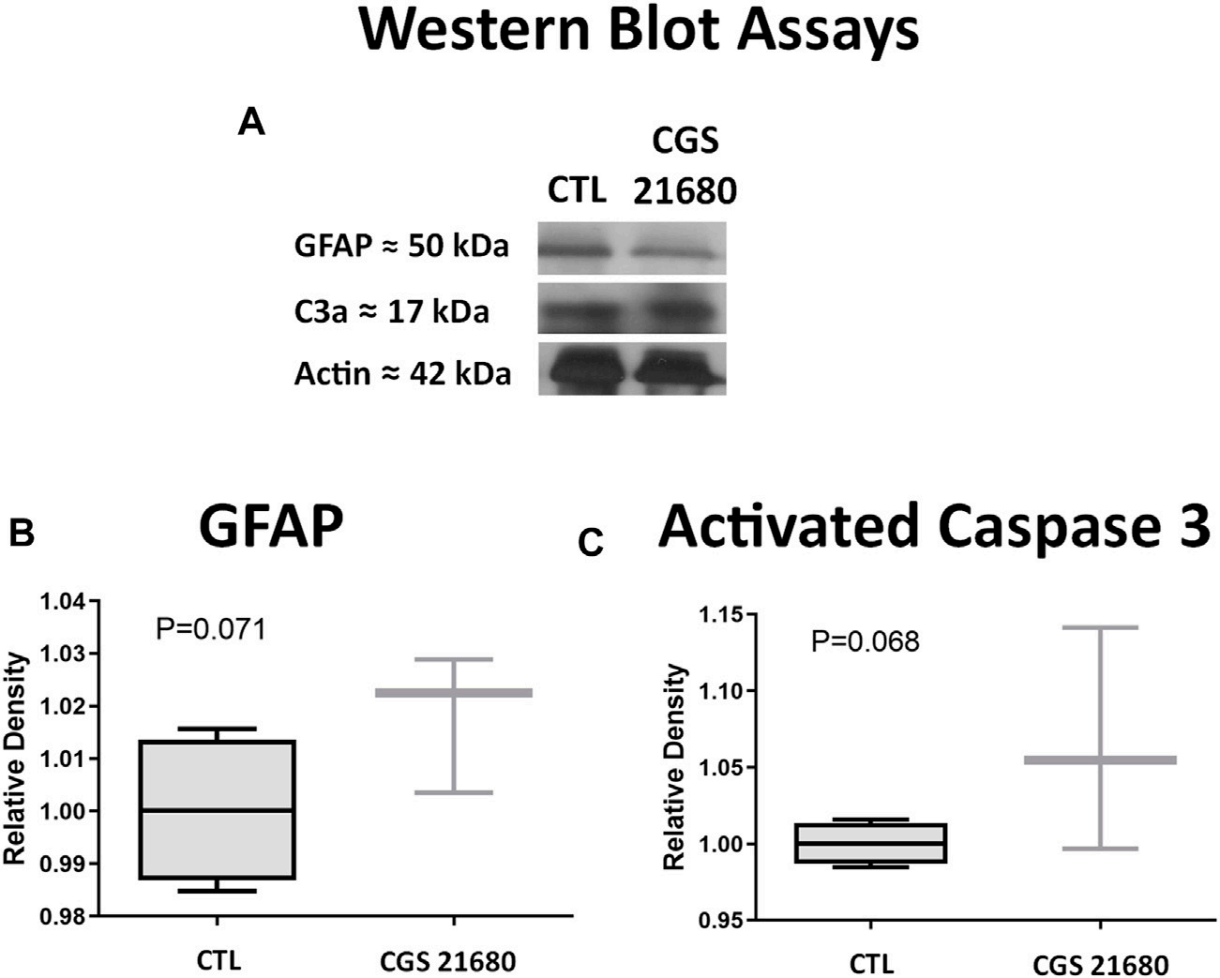
**(A)** Western blots of retinas from CGS21680-treated and CTL eyes and their quantifications. From top to bottom, bands correspond to GFAP, activated Caspase-3, and actin. **(B)** Quantification of GFAP by WB. **(C)** Quantification of activated caspase-3 by WB. Relative densities were normalized against CTL β-actin. Boxes represent 25 and 75 percentiles, whiskers represent minimum and maximum values, and transverse lines represent medians. No statistical differences were found between groups.

No significant differences were seen in protein expression of GFAP and activated caspase-3 between the retinas of eyes treated with CGS21680 and CTL (Figure 2 and Supplementary Figure S4).

#### Electroretinography After Administration of CGS21680

**FIGURE 3:**
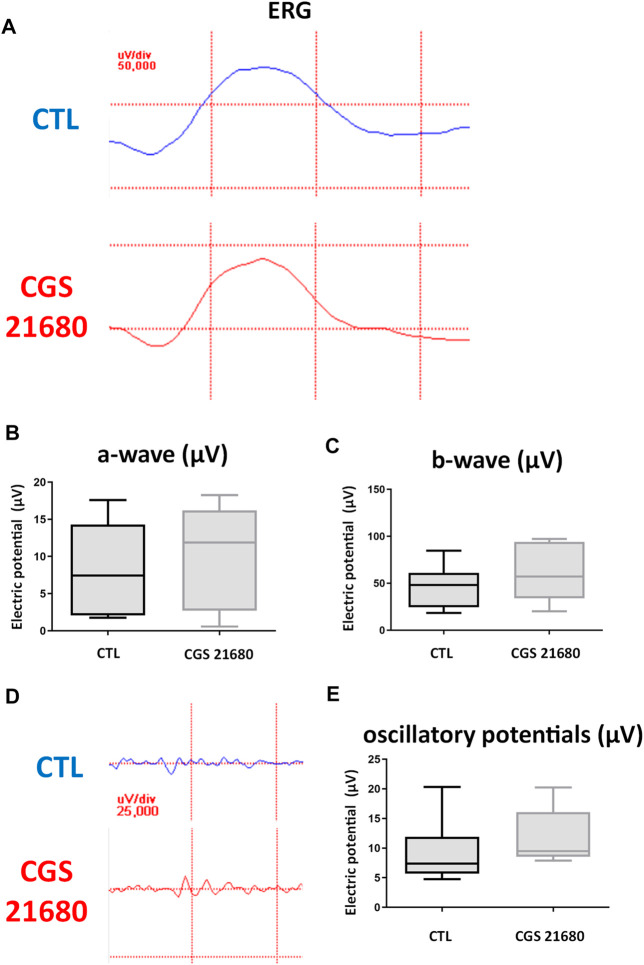
ERG recordings of control eyes (CTL) and CGS21680-treated eyes. **(A)** ERG recording a CTL eye (above) and a CGS21680-treated eye (below) 1 week after CI. **(B)** Quantification of a-wave. **(C)** Quantification of b-wave. **(D)** Response of oscillatory potentials 1 week after exposure to CI of a CTL eye (above) and an eye treated with CGS21680 (below). **(E)** Quantifications of OP. Boxes represent 25 and 75 percentiles, whiskers represent minimum and maximum values, and transverse lines represent medians. No statistical differences were found between groups.

The eyes treated with CGS21680 did not show significant differences in any of the studied parameters between treated eyes 7 days post-illumination and control eyes 7 days post-illumination: a-wave (*p* = 0.27), b-wave (*p* = 0.19), and oscillatory potentials (*p* = 0.22, Table 2 and Figure 3).

**TABLE 2:** ERG recordings of eyes treated with CGS21680 and their controls. Mean values and standard deviations are shown. No statistical differences were found between groups.

	Control		CGS 21680	
	Basal	7 days Post-CI	Basal	7 days Post-CI
a-Wave (µV)	3.68 ± 1.88	8.28 ± 6.2	4.64 ± 1.71	10.25 ± 6.91
b-Wave (µV)	99.27 ± 41.82	46.53 ± 23.46	120.9 ± 60.93	60.48 ± 30.19
OP (µV)	37.05 ± 18.11	9.15 ± 5.71	42.71 ± 21.51	11.74 ± 5.71

#### Effects of CGS21680 on Gene Expression (Quantitative Reverse Transcription-Polymerase Chain Reaction)

**FIGURE 4:**
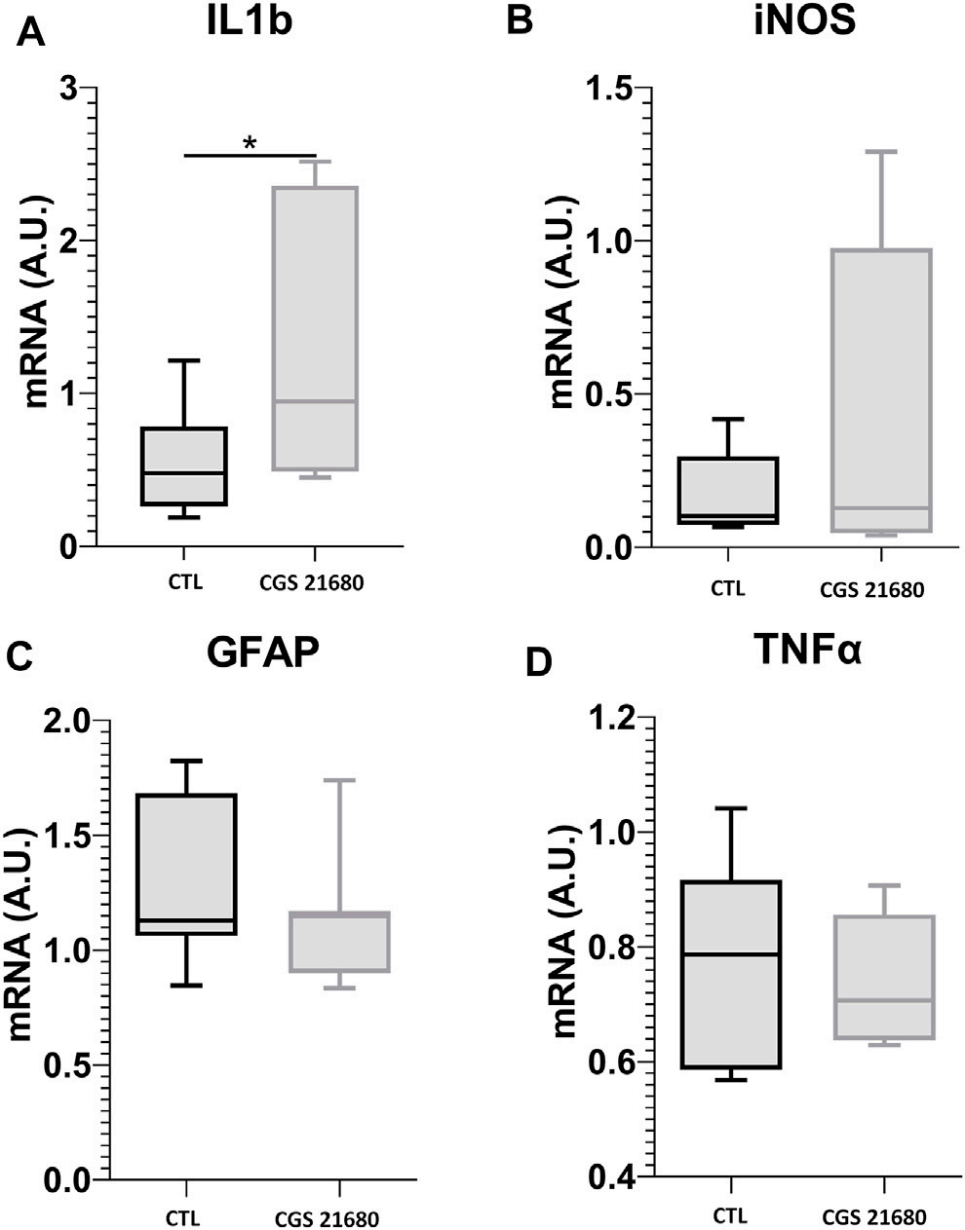
qRT-PCR for IL-1β **(A)**, iNOS **(B)**, GFAP **(C)**, and TNF-α **(D)** mRNAs in retinas from CGS21680 and their respective CTL. Boxes represent 25 and 75 percentiles, whiskers represent minimum and maximum values, and transverse lines represent medians (Student’s *t*-test, *p < 0.05).

To investigate the mechanism of action of the A2AR agonist, CGS21680, we studied the expression of genes involved in cell damage and inflammation. qRT-PCRs of the retinas were performed after the treatment with CGS21680, followed by 24 h of CI. Cytokine IL-1β expression increased significantly (1.278 ± 0.9059 *vs*. 0.5573 ± 0.3511; *p* < 0.05) (Figure 4A), whereas inducible nitric oxide synthase (iNOS) messenger RNA (mRNA) and TNF-α mRNA did not change significantly (Figures 4B,D). The astroglial marker, GFAP mRNA, did not change after treatment with CGS21680 (Figure 4C).

### Effects of the Administration of SCH58261 Before Light-Induced Retinal Degeneration

#### Effects on Photoreceptor Apoptosis and Gliosis

**FIGURE 5:**
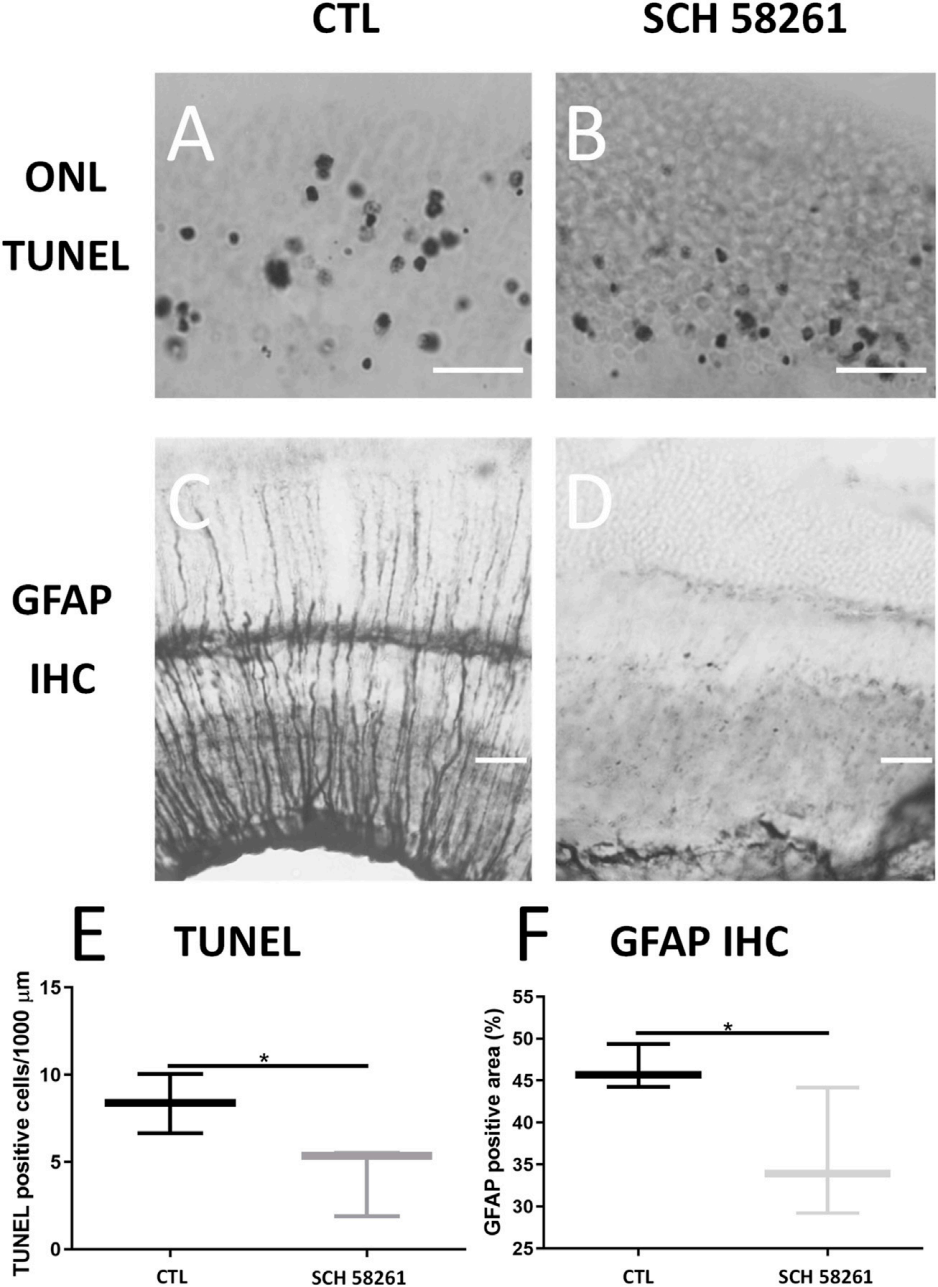
Treatment with SCH58261 decreased number of apoptotic nuclei and GFAP immunoreactive areas. **(A,B)** Representative section showing TUNEL staining of ONL of retina of a CTL eye **(A)** and an SCH58261-treated eye **(B)**. Treated eyes had a lower number of apoptotic nuclei than controls. Scale bar: 20 μm. **(C,D)** GFAP-immunostained section from retina of a CTL eye **(C)** and an SCH58261-treated eye **(D)**. Less GFAP immunoreactivity of Müller cells is observed in retina of SCH58261-treated eye compared with CTL. **(E)**. Quantification of TUNEL-positive ONL cells. SCH58261 produced a significant decrease in number of positive nuclei in ONL compared with CTL. Student’s *t*-test, *p < 0.05. **(F)** Quantification of GFAP immunoreactive area. SCH58261 produced a significant decrease in GFAP expression compared with CTL. Boxes represent 25 and 75 percentiles, whiskers represent minimum and maximum values, and transverse lines represent medians.

TUNEL staining showed that the number of positive nuclei decreased in SCH58261-treated eyes, indicating a lower number of apoptotic photoreceptors (8.354 ± 1.701 *vs*. 4.247 ± 2.056, *p* < 0.05) (Figures 5A,B,E and Supplementary Figure S3).

The quantification of GFAP immunoreactivity showed the reduction of GFAP-immunostained areas in the retinas of SCH58261-treated eyes, demonstrating less glial activation (35.76 ± 7.625% *vs*. 46.44 ± 2.643%, *p* < 0.05). (Figures 5C,D,F).

#### Effects of SCH58261 on Apoptosis and Glial Reactivity by Western Blot

**FIGURE 6:**
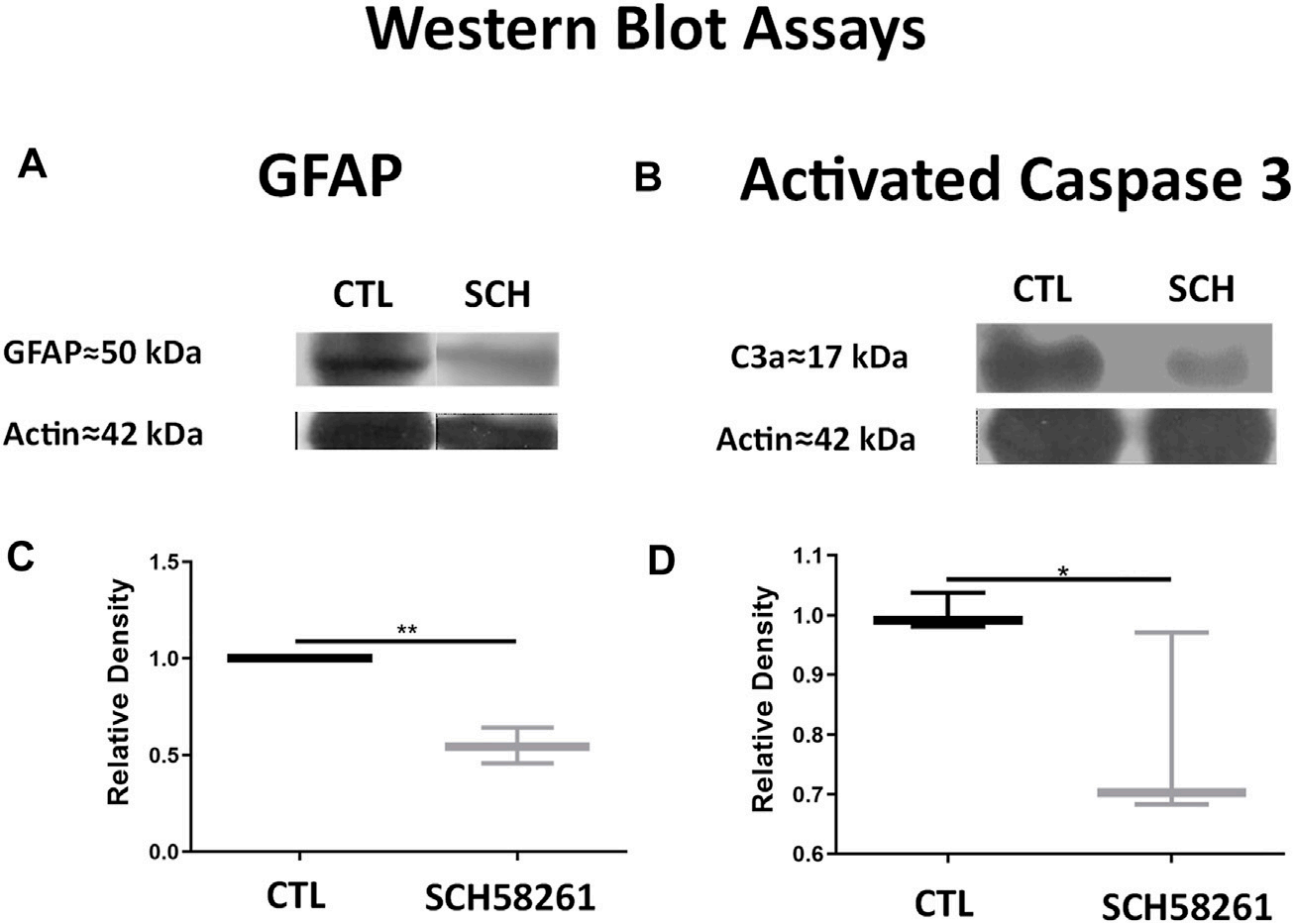
Treatment with SCH58261 decreased levels of GFAP and activated caspase-3. **(A)** Representative Western blot (WB) for GFAP of CTL and SCH58261-treated eyes. **(B)** Representative Western blot for activated caspase-3 of CTL and SCH58261-treated eyes. **(C)** Quantifications of GFAP WB of CTL and SCH58261-treated eyes **(D)**. Quantification of activated caspase-3 WB of CTL and SCH58261-treated eyes. Relative densities were normalized against CTL β-actin. Boxes represent 25 and 75 percentiles, whiskers represent minimum and maximum values, and transverse lines represent medians. Student’s *t*-test, *p < 0.05; **p < 0.01.

Changes in the levels of activated caspase-3 and GFAP are in agreement with previous TUNEL and IHC results. A significant decrease of GFAP levels in the treated eyes was found (0.5477 ± 0.09308 *vs*. 1 ± 0.06348, *p* < 0.01) (Figures 6A,C and Supplementary Figure S4). Also, a statistically significant decrease in activated caspase-3 levels in the retinas of SCH58261-treated eyes was confirmed (0.7853 ± 0.1611 *vs*. 1 ± 0.030220, *p* < 0.05) (Figures 6B,D and Supplementary Figure S4).

#### Effects of SCH58261 on Retinal Function Determined by Electroretinography

**FIGURE 7:**
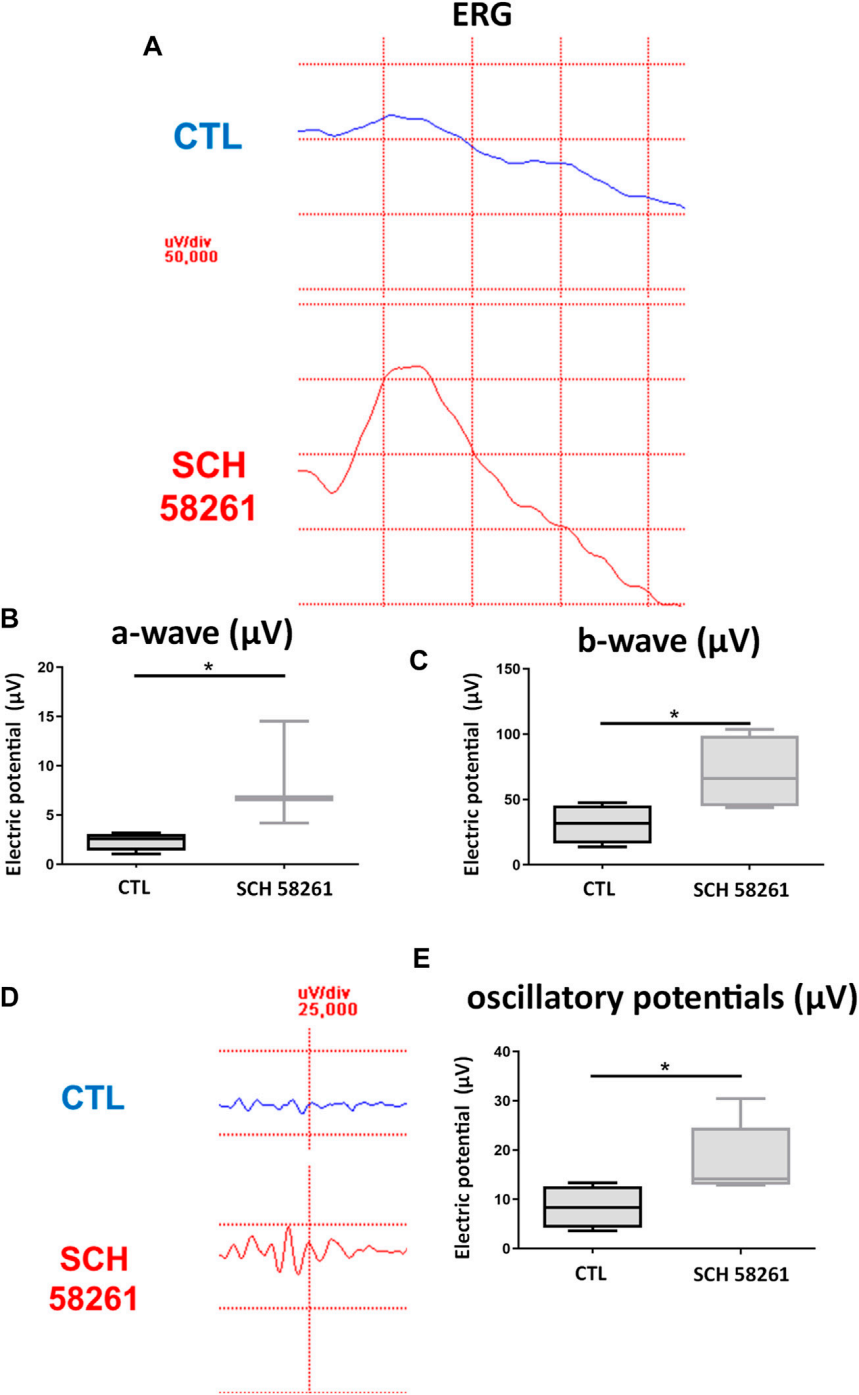
Effect of treatment with SCH58261 on ERG recordings post CI. **(A)** ERG recording 1 week after exposure to 24 h of CI of a CTL eye (above) and an SCH58261-treated eye (below). **(B)** Quantification of a-wave. **(C)** Quantification of b-wave. **(D)** Response of oscillatory potentials 1 week after 24 h of CI of a CTL eye (above) and an SCH58261-treated eye (below). **(E)** Quantification of OP. Boxes represent 25 and 75 percentiles, whiskers represent minimum and maximum values, and transverse lines represent medians. Student’s *t*-test,*p < 0.05.

SCH58261-treated eyes showed a greater response of the photoreceptors as larger a-wave was recorded compared with CTL eyes (*p* < 0.05) (Figures 7A,B; Table 3).

**TABLE 3:** ERG recordings and oscillatory potentials of eyes treated with SCH58261 and their controls. Mean values and standard deviations are shown, Student's *t*-test,*p < 0.05.

	Control		SCH 58261	
	Basal	Post-CI	Basal	Post-CI
a-Wave (µV)	4.62 ± 3.56	2.35 ± 0.92	4.84 ± 4.40	8.46 ± 5.39*
b-Wave (µV)	99.79 ± 50.97	31.23 ± 14.98	99.02 ± 52.35	69.98 ± 28.88*
OP (µV)	33.27 ± 10.35	8.405 ± 4.363	37.59 ± 15.17	17.83 ± 7.41*

Also, the function of the inner retina was protected, as the amplitude of the b-wave and OP was significantly larger in SCH58261-treated eyes compared with CTL eyes (*p* < 0.05 in both cases) (Figures 7C–E; Table 3).

#### Effects of SCH58261 on Gene Expression (Quantitative Reverse Transcription-Polymerase Chain Reaction)

**FIGURE 8:**
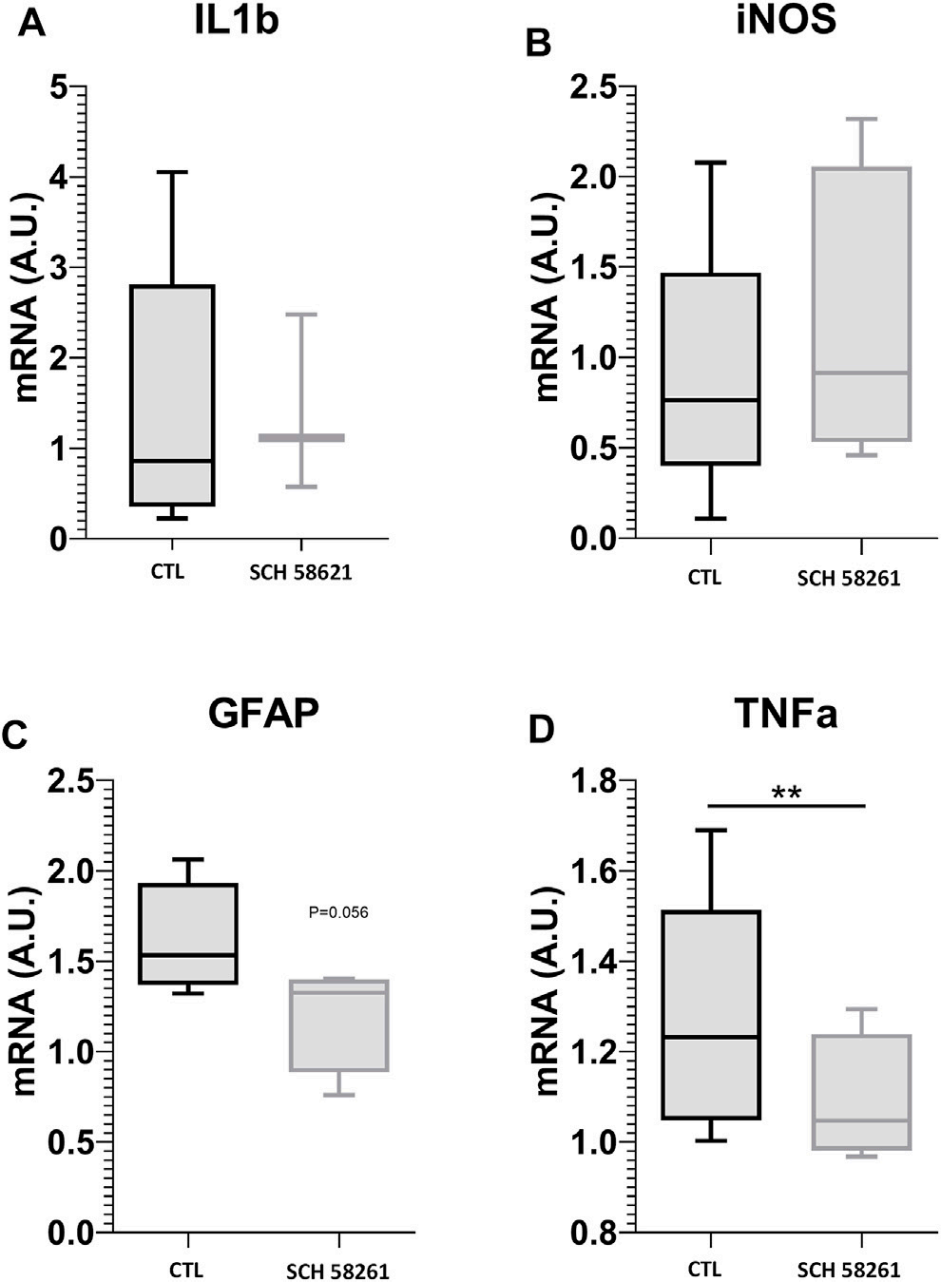
qRT-PCR for IL-1β **(A)**, iNOS **(B)**, GFAP **(C)**, and TNF-α **(D)** mRNAs in retinas from SCH58261 and their respective CTL. Boxes represent 25 and 75 percentiles, whiskers represent minimum and maximum values, and transverse lines represent medians. Student’s *t*-test, *p < 0.05; **p < 0.01.

Similarly, to investigate the neuroprotective mechanism of the A2AR antagonist SCH58261, we studied the expression of genes involved in cell damage and inflammation. qRT-PCRs of the retinas were performed after the treatment with SCH58261, followed by 24 h of CI. TNF-α decreased significantly in SCH58261-treated retinas (1.089 ± 0.1431 *vs*. 1.271 ± 0.2668, *p* < 0.01) (Figure 8D), whereas the expression of cytokine IL-1β, iNOS, and GFAP did not change significantly (Figures 8A–C).

## Discussion

Results presented herein indicated that an A2AR agonist (CGS21680) exacerbated the damage induced by light exposure to the retina. CGS21680-treated eyes presented higher densities of apoptotic nuclei in ONL and higher glial reactivity, evidenced by an increase of the GFAP immunoreactive area. However, the WB study did not confirm these data, and functional studies using ERG showed no differences between treated eyes and their CTL. This phenomenon could be explained because CI produced such functional damage that it cannot be worsened by an A2AR agonist. Also, it may be speculated that the techniques used to detect apoptosis (TUNEL) and GFAP immunohistochemistry have greater sensitivity to detect damage at the cellular level than the WB of the full retinal tissue. Alternatively, it must be mentioned that there are strong pharmacological arguments in brain tissue questioning the selectivity of CGS21680, as it could also act as an agonist for the A1 receptor (Zhang et al., 1994; Cunha et al., 1996; Cunha et al., 1999). This could also be the reason underlying the lack of effect of this drug.

On the opposite, we found that a lower A2AR activity due to an antagonist intravitreal injection was protective to the retina, as SCH58261-treated retinas showed smaller amounts of apoptotic nuclei in ONL and lower glial reactivity. Both data were confirmed by WB because SCH58261-treated retinas had lower levels of activated caspase-3 and GFAP. Finally, SCH 58261 also protected the retinal function, as all ERG parameters were preserved.

It was demonstrated that A2AR inhibitors provide important protective mechanisms in the CNS, as low concentrations of adenosine activate A1R and inhibit the release of excitatory amino acids, but higher concentrations of adenosine activate A2AR and block A1R through a receptor–receptor allosteric trans-inhibition (Ciruela et al., 2006).

The role of adenosine in the inflammatory response to retinal injury is well known, and it was reported that SCH58261 protects from photoreceptor loss because it prevents the upregulation of proinflammatory mediators and the alterations of the complement system in microglial cells (Madeira et al., 2018). Also, KW6002, another A2AR antagonist, reduced the inflammatory microglial response and protected the retina from ischemic injury and reperfusion (Boia et al., 2017). Caffeine, an unspecific A2AR antagonist, was neuroprotective, as it lowered intraocular pressure and reduced the activation of microglia and the inflammatory cytokines IL-1β and TNF-α in a mouse glaucoma model (Madeira et al., 2016b). To investigate the neuroprotective mechanism of the A2AR antagonist, we studied the expression of genes involved in inflammation and cell damage by qRT-PCR, and we found that SCH58261 lowered the levels of TNF-α expression, supporting the idea that it reduces the upregulation of inflammatory microglial mediators. This lower inflammatory milieu could favor the survival of the photoreceptors and could allow the conservation of the function.

The observed neuroprotective effect of A2AR antagonism in LIRD is aligned with the neuroprotective effect of chronic caffeine intake (Cunha and Agostinho, 2010) and with caffeine reduction of apoptosis in oxygen-induced retinopathy (Zhang et al., 2017).

On the contrary, using two different models of perinatal brain injury, CGS21680 produced a partial increase of some microglial cytokines such as IL-1β and TNF-α, as well as an increase of iNOS (Colella et al., 2018). In agreement with this, in our model of CI, CGS21680 produced a similar phenomenon, as we also detected an increase of the cytokine IL-1β. Surprisingly, no significant effects were observed in iNOS and TNF-α mRNA expression.

Herein, we detected lower levels of Müller glia activation in SCH58261-treated eyes, suggesting that modulation by A2AR may be involved in the inflammatory reaction. This finding is in line with the observation that the injection of antagonist SCH 442416 reverses the changes in the expression of channels and transporters in Müller cells responsible for maintaining retinal homeostasis (Yang et al., 2015).

Although the role of A2AR antagonist on microglial inflammation seems to be the main mechanism involved in the protection of the retina, we postulate that a direct neuroprotective effect on the photoreceptors themselves cannot be ruled out, as A2 receptors are localized in rabbit and mouse outer retinas (Blazynski, 1990; Blazynski and Perez, 1991), and A2AR was also found in the photoreceptors of different species (McIntosh and Blazynski, 1994; Rey and Burnside, 1999; Stella et al., 2002; Li et al., 2014). Further studies are needed to confirm this hypothesis.

Finally, we studied the changes in the expression of the A2A receptor. CGS 21680 produced no significant changes (Supplementary Figure S1), but SCH 58261 produced a significant reduction in the expression of the receptor (Supplementary Figure S2). This would increase the antagonistic effects of SCH 58261 by diminishing the available receptors and may also explain the protective effect of the drug.

In summary, this study shows that the blockade of A2A receptors before exposure to continuous light prevents retina damage and preserves retinal function by lowering inflammation, glial reactivity, and apoptosis in ONL. These results allow us to postulate that the modulation of A2AR activity may be a strategy that deserves to be evaluated in degenerative pathologies of the retina.

## Data Availability

The original contributions presented in the study are included in the article/Supplementary Material; further inquiries can be directed to the corresponding author.

## Supplementary Material

SUPPLEMENTARY FIGURE S1 qRT-PCR for A2A receptor in retinas from CGS 21680 treated eyes and their respective CTL. Primers A2A: Forward CGG​GAA​CTC​CAC​GAA​GAC​C Reverse AGC​AAA​GAG​CCC​GAC​GAT​G. Boxes represent 25 and 75 percentiles, whiskers represent minimum and maximum values and transverse lines represent means.

SUPPLEMENTARY FIGURE S2 qRT-PCR for A2A receptor in retinas from SCH58261 treated eyes and their respective CTL. Primers A2A: Forward CGG​GAA​CTC​CAC​GAA​GAC​C Reverse AGC​AAA​GAG​CCC​GAC​GAT​G. Boxes represent 25 and 75 percentiles, whiskers represent minimum and maximum values and transverse lines represent means. Student’s *t* test, * P <0.05.

SUPPLEMENTARY FIGURE S3 Left. Representative section of a retina treated with SCH58261 and the corresponding control. Observe the lower number of apoptotic nuclei in ONL. Right: Representative section of a retina treated with CGS21680 and the corresponding control. PRL, photoreceptor layer; ONL, outer nuclear layer; OPL, outer plexiform layer; INL, inner nuclear layer, IPL, inner plexiform layer; GCL, ganglion cell layer. Scale bar: 20 μm.

SUPPLEMENTARY FIGURE S4 Top. Western blot membranes of retinas from CGS21680-treated and CTL eyes. From top to bottom, the bands correspond to activated caspase-3, GFAP and actin. B. Bottom. Western blot membranes of retinas from SCH 58261-treated and CTL eyes. From top to bottom, the bands correspond to activated Caspase-3, GFAP and actin. B.

## Glossary

A1R: adenosine receptor type A1
A2AR: adenosine receptor type A2A
Cat: catalog
CI: continuous illumination
CNS: central nervous system
Co.: Company
CTL: control
DNAseI: deoxyribonuclease
ECL: Enhanced Chemiluminiscence
ERG: electroretinography
GCL: ganglion cell layer
GE: General Electric
GFAP: glial fibrillary acidic protein
h: hour
HRP: horseradish peroxidase
IHC: immunohistochemistry
HP: Hewlett Packard
Hz: Hertz
IL: illuminated
IL-1β: interleukin-1β
IL-6: interleukin-6
INL: inner nuclear layer
iNOS: inducible nitric oxide synthase
IPL: inner plexiform layer
IR: immunoreactive
LIRD: light-induced retinal degeneration
µ: micron
µV: microvolt
mRNA: messenger ribonucleic acid
NFL: nerve fiber layer
NIH: National Institutes of Health
OD: optical density
ONL: outer nuclear layer
OP: oscillatory potential
OPL: outer plexiform layer
PCR: polymerase chain reaction
PHL: photoreceptor layer
qRT-PCR: quantitative reverse transcription polymerase chain reaction
RNA: Ribonucleic Acid
ROI: region of interest
RPE: retinal pigment epithelium
RT: room temperature
TNF-α: tumor necrosis factor-α
TUNEL: terminal deoxynucleotidyltransferase dUTP nick end labeling
V: Volt
W: Watt
WB: Western Blot
w/v: weight/volume
18S: 18 Svedberg

## References

[B2] Blazynski C. (1990). Discrete Distributions of Adenosine Receptors in Mammalian Retina. J. Neurochem. 54, 648–655. 10.1111/j.1471-4159.1990.tb01920.x 2299359

[B3] Blazynski C. Perez M. T. (1991). Adenosine in Vertebrate Retina: Localization, Receptor Characterization, and Function. Cell Mol Neurobiol. 11, 463–484. 10.1007/BF00734810 1683815 PMC11567418

[B4] Boeck C. R. Kroth E. H. Bronzatto M. J. Vendite D. (2005). Adenosine Receptors Co-operate with NMDA Preconditioning to Protect Cerebellar Granule Cells against Glutamate Neurotoxicity. Neuropharmacology 49, 17–24. 10.1016/j.neuropharm.2005.01.024 15992577

[B5] Boia R. Elvas F. Madeira M. H. Ares I. D. Rodrigues-Neves A. C. Tralhao P. (2017). Treatment with A2A Receptor Antagonist KW6002 and Caffeine Intake Regulate Microglia Reactivity and Protect Retina against Ischemia Damage. Cell Death Dis 8 (10), e3065. 10.1038/cddis.2017.451 28981089 PMC5680573

[B6] Canas P. M. Porciúncula L. O. Cunha G. M. Silva C. G. Machado N. J. Oliveira J. M. (2009). Adenosine A2A Receptor Blockade Prevents Synaptotoxicity and Memory Dysfunction Caused by Beta-Amyloid Peptides via P38 Mitogen-Activated Protein Kinase Pathway. J. Neurosci. 29, 14741–14751. 10.1523/JNEUROSCI.3728-09.2009 19940169 PMC6665997

[B7] Ciruela F. Casadó V. Rodrigues R. J. Luján R. Burgueño J. Canals M. (2006). Presynaptic Control of Striatal Glutamatergic Neurotransmission by Adenosine A1-A2a Receptor Heteromers. J. Neurosci. 26, 2080–2087. 10.1523/JNEUROSCI.3574-05.2006 16481441 PMC6674939

[B8] Colella M. Zinni M. Pansiot J. Cassanello M. Mairesse J. Ramenghi L. (2018). Modulation of Microglial Activation by Adenosine A2a Receptor in Animal Models of Perinatal Brain Injury. Front. Neurol. 9, 605. 10.3389/fneur.2018.00605 30254599 PMC6141747

[B9] Cunha R. A. (2016). How Does Adenosine Control Neuronal Dysfunction and Neurodegeneration? J. Neurochem. 139, 1019–1055. 10.1111/jnc.13724 27365148

[B10] Cunha R. A. Agostinho P. M. (2010). Chronic Caffeine Consumption Prevents Memory Disturbance in Different Animal Models of Memory Decline. J. Alzheimers Dis. 20 (Suppl. 1), S95–S116. 10.3233/JAD-2010-1408 20182043

[B11] Cunha R. A. Constantino M. D. Ribeiro J. A. (1999). G Protein Coupling of CGS 21680 Binding Sites in the Rat hippocampus and Cortex Is Different from that of Adenosine A1 and Striatal A2A Receptors. Naunyn Schmiedebergs Arch. Pharmacol. 359, 295–302. 10.1007/pl00005355 10344528

[B12] Cunha R. A. Johansson B. Constantino M. D. Sebastião A. M. Fredholm B. B. (1996). Evidence for High-Affinity Binding Sites for the Adenosine A2A Receptor Agonist [3H] CGS 21680 in the Rat hippocampus and Cerebral Cortex that Are Different from Striatal A2A Receptors. Naunyn Schmiedebergs Arch. Pharmacol. 353, 261–271. 10.1007/BF00168627 8692280

[B13] Dos Santos-Rodrigues A. Pereira M. R. Brito R. de Oliveira N. A. Paes-de-Carvalho R. (2015). Adenosine Transporters and Receptors: Key Elements for Retinal Function and Neuroprotection. Vitam Horm. 98, 487–523. 10.1016/bs.vh.2014.12.014 25817878

[B14] Dureau P. Bonnel S. Menasche M. Dufier J. L. Abitbol M. (2001). Quantitative Analysis of Intravitreal Injections in the Rat. Curr. Eye Res. 22, 74–77. 10.1076/ceyr.22.1.74.6974 11402382

[B15] Font L. Mingote S. Farrar A. M. Pereira M. Worden L. Stopper C. (2008). Intra-accumbens Injections of the Adenosine A2A Agonist CGS 21680 Affect Effort-Related Choice Behavior in Rats. Psychopharmacology (Berl) 199, 515–526. 10.1007/s00213-008-1174-z 18491078 PMC2643064

[B16] Gomes C. A. Simões P. F. Canas P. M. Quiroz C. Sebastião A. M. Ferré S. (2009). GDNF Control of the Glutamatergic Cortico-Striatal Pathway Requires Tonic Activation of Adenosine A Receptors. J. Neurochem. 108, 1208–1219. 10.1111/j.1471-4159.2009.05876.x 19141075 PMC2676206

[B17] Gori M. B. Girardi E. (2013). 3-Mercaptopropionic Acid-Induced Repetitive Seizures Increase GluN2A Expression in Rat hippocampus: a Potential Neuroprotective Role of Cyclopentyladenosine. Cel Mol Neurobiol. 33, 803–813. 10.1007/s10571-013-9947-2 PMC11497892 23748434

[B18] Grishagin I. V. (2015). Automatic Cell Counting with ImageJ. Anal. Biochem. 473, 63–65. 10.1016/j.ab.2014.12.007 25542972

[B19] Gyoneva S. Shapiro L. Lazo C. Garnier-Amblard E. Smith Y. Miller G. W. (2014). Adenosine A2A Receptor Antagonism Reverses Inflammation-Induced Impairment of Microglial Process Extension in a Model of Parkinson's Disease. Neurobiol. Dis. 67, 191–202. 10.1016/j.nbd.2014.03.004 24632419 PMC4072497

[B20] Hancock M. B. (1984). Visualization of Peptide-Immunoreactive Processes on Serotonin-Immunoreactive Cells Using Two-Color Immunoperoxidase Staining. J. Histochem. Cytochem. 32, 311–314. 10.1177/32.3.6198359 6198359

[B21] Housley G. D. Bringmann A. Reichenbach A. (2009). Purinergic Signaling in Special Senses. Trends Neurosci. 32, 128–141. 10.1016/j.tins.2009.01.001 19232752

[B22] Jenner P. (2014). An Overview of Adenosine A2A Receptor Antagonists in Parkinson's Disease. Int. Rev. Neurobiol. 119, 71–86. 10.1016/B978-0-12-801022-8.00003-9 25175961

[B23] Kaster M. P. Machado N. J. Silva H. B. Nunes A. Ardais A. P. Santana M. (2015). Caffeine Acts through Neuronal Adenosine A2A Receptors to Prevent Mood and Memory Dysfunction Triggered by Chronic Stress. Proc. Natl. Acad. Sci. U S A. 112, 7833–7838. 10.1073/pnas.1423088112 26056314 PMC4485143

[B24] Leibovich S. J. Chen J. F. Pinhal-Enfield G. Belem P. C. Elson G. Rosania A. (2002). Synergistic Up-Regulation of Vascular Endothelial Growth Factor Expression in Murine Macrophages by Adenosine A(2A) Receptor Agonists and Endotoxin. Am. J. Pathol. 160, 2231–2244. 10.1016/S0002-9440(10)61170-4 12057925 PMC1850844

[B25] Li B. Rosenbaum P. S. Jennings N. M. Maxwell K. M. Roth S. (1999). Differing Roles of Adenosine Receptor Subtypes in Retinal Ischemia-Reperfusion Injury in the Rat. Exp. Eye Res. 68, 9–17. 10.1006/exer.1998.0573 9986737

[B26] Li H. Chuang A. Z. O'Brien J. (2014). Regulation of Photoreceptor gap junction Phosphorylation by Adenosine in Zebrafish Retina. Vis. Neurosci. 31, 237–243. 10.1017/S095252381300062X 24844306 PMC4109651

[B27] Madeira M. H. Boia R. Elvas F. Martins T. Cunha R. A. Ambrósio A. F. (2016a). Selective A2A Receptor Antagonist Prevents Microglia-Mediated Neuroinflammation and Protects Retinal Ganglion Cells from High Intraocular Pressure-Induced Transient Ischemic Injury. Transl Res. 169, 112–128. 10.1016/j.trsl.2015.11.005 26685039

[B28] Madeira M. H. Ortin-Martinez A. Nadal-Nícolas F. Ambrósio A. F. Vidal-Sanz M. Agudo-Barriuso M. (2016b). Caffeine Administration Prevents Retinal Neuroinflammation and Loss of Retinal Ganglion Cells in an Animal Model of Glaucoma. Sci. Rep. 6, 27532. 10.1038/srep27532 27270337 PMC4897621

[B29] Madeira M. H. Rashid K. Ambrósio A. F. Santiago A. R. Langmann T. (2018). Blockade of Microglial Adenosine A2A Receptor Impacts Inflammatory Mechanisms, Reduces ARPE-19 Cell Dysfunction and Prevents Photoreceptor Loss *In Vitro* . Sci. Rep. 8, 2272. 10.1038/s41598-018-20733-2 29396515 PMC5797099

[B30] McIntosh H. H. Blazynski C. (1994). Characterization and Localization of Adenosine A2 Receptors in Bovine Rod Outer Segments. J. Neurochem. 62, 992–997. 10.1046/j.1471-4159.1994.62030992.x 8113818

[B31] Nobre H. V. Cunha G. M. de Vasconcelos L. M. Magalhães H. I. Oliveira Neto R. N. Maia F. D. (2010). Caffeine and CSC, Adenosine A2a Antagonists, Offer Neuroprotection against 6-OHDA-Induced Neurotoxicity in Rat Mesencephalic Cells. Neurochem. Int. 56, 51–58. 10.1016/j.neuint.2009.09.001 19782116

[B32] Ongini E. (1998). SCH58261: A Selective A2A Adenosine Receptor Antagonist. Drug Dev. Res. 42, 63–70.

[B33] Ostwald P. Park S. S. Toledano A. Y. Roth S. (1997). Adenosine Receptor Blockade and Nitric Oxide Synthase Inhibition in the Retina: Impact upon post-ischemic Hyperemia and the Electroretinogram. Vis. Res. 37, 3453–3461. 10.1016/S0042-6989(96)00222-2 9425522

[B34] Paterniti I. Melani A. Cipriani S. Corti F. Mello T. Mazzon E. (2011). Selective Adenosine A2a Receptor Agonists and Antagonists Protect against Spinal Cord Injury through Peripheral and central Effects. J. Neuroinflammation 8, 31. 10.1186/1742-2094-8-31 21486435 PMC3096915

[B35] Perígolo-Vicente R. Ritt K. Gonçalves-de-Albuquerque C. F. Castro-Faria-Neto H. C. Paes-de-Carvalho R. Giestal-de-Araujo E. (2014). IL-6, A1 and A2AR: a Crosstalk that Modulates BDNF and Induces Neuroprotection. Biochem. Biophys. Res. Commun. 449, 477–482. 10.1016/j.bbrc.2014.05.036 24845382

[B36] Rey H. L. Burnside B. (1999). Adenosine Stimulates Cone Photoreceptor Myoid Elongation via an Adenosine A2-like Receptor. J. Neurochem. 72, 2345–2355. 10.1046/j.1471-4159.1999.0722345.x 10349843

[B37] Rosim F. E. Persike D. S. Nehlig A. Amorim R. P. de Oliveira D. M. Fernandes M. J. (2011). Differential Neuroprotection by A(1) Receptor Activation and A(2A) Receptor Inhibition Following Pilocarpine-Induced Status Epilepticus. Epilepsy Behav. 22, 207–213. 10.1016/j.yebeh.2011.07.004 21852200

[B38] Roth S. Rosenbaum P. S. Osinski J. Park S. S. Toledano A. Y. Li B. (1997). Ischemia Induces Significant Changes in Purine Nucleoside Concentration in the Retina-Choroid in Rats. Exp. Eye Res. 65, 771–779. 10.1006/exer.1997.0391 9441700

[B39] Santiago A. R. Baptista F. I. Santos P. F. Cristóvão G. Ambrósio A. F. Cunha R. A. (2014). Role of Microglia Adenosine A2A Receptors in Retinal and Brain Neurodegenerative Diseases. Mediators Inflamm. 2014, 465694. 10.1155/2014/465694 25132733 PMC4124703

[B40] Severns M. L. Johnson M. A. Bresnick G. H. (1994). Methodologic Dependence of Electroretinogram Oscillatory Potential Amplitudes. Doc Ophthalmol. 86, 23–31. 10.1007/BF01224625 7956683

[B41] Soliño M. López E. M. Rey-Funes M. Loidl C. F. Larrayoz I. M. Martínez A. (2018). Adenosine A1 Receptor: A Neuroprotective Target in Light Induced Retinal Degeneration. PLoS One 13 (6), e0198838. 10.1371/journal.pone.0198838 29912966 PMC6005487

[B42] Stella S. L. Bryson E. J. Thoreson W. B. (2002). A2 Adenosine Receptors Inhibit Calcium Influx through L-type Calcium Channels in Rod Photoreceptors of the Salamander Retina. J. Neurophysiol. 87, 351–360. 10.1152/jn.00010.2001 11784755

[B43] Stone T. W. Ceruti S. Abbracchio M. P. (2009). Adenosine Receptors and Neurological Disease: Neuroprotection and Neurodegeneration. Handb Exp. Pharmacol. 193, 535–587. 10.1007/978-3-540-89615-9_17 19639293

[B44] Tebano M. T. Martire A. Chiodi V. Ferrante A. Popoli P. (2010). Role of Adenosine A(2A) Receptors in Modulating Synaptic Functions and Brain Levels of BDNF: a Possible Key Mechanism in the Pathophysiology of Huntington's Disease. ScientificWorldJournal 10, 1768–1782. 10.1100/tsw.2010.164 20842321 PMC5763899

[B45] Yang Z. Huang P. Liu X. Huang S. Deng L. Jin Z. (2015). Effect of Adenosine and Adenosine Receptor Antagonist on Müller Cell Potassium Channel in Rat Chronic Ocular Hypertension Models. Sci. Rep. 5, 11294. 10.1038/srep11294 26063641 PMC4462755

[B46] Zhang G. Franklin P. H. Murray T. F. (1994). Activation of Adenosine A1 Receptors Underlies Anticonvulsant Effect of CGS21680. Eur. J. Pharmacol. 255, 239–243. 10.1016/0014-2999(94)90104-x 8026549

[B47] Zhang S. Zhou R. Li B. Li H. Wang Y. Gu X. (2017). Caffeine Preferentially Protects against Oxygen-Induced Retinopathy. FASEB J. 31, 3334–3348. 10.1096/fj.201601285R 28420694 PMC6207216

