# Supplementary figures and images for: Adenosine A2A Receptor: A New Neuroprotective Target in Light-Induced Retinal Degeneration
Source: Front Pharmacol. 2022 Mar 21;13:840134. doi: 10.3389/fphar.2022.840134 (PMC8977837; doi:10.3389/fphar.2022.840134)

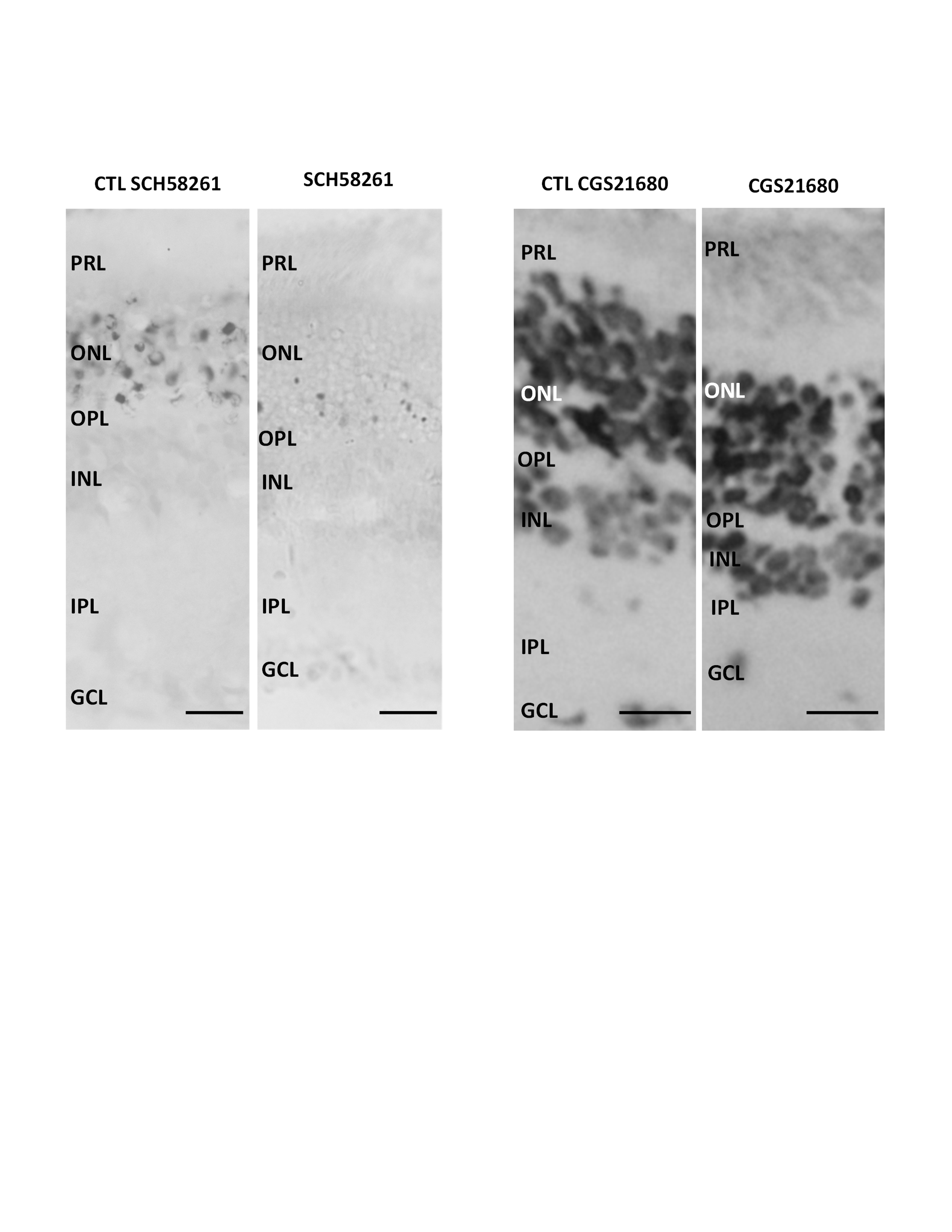

Supplement: Supplementary file 1 [file Image3.TIF]

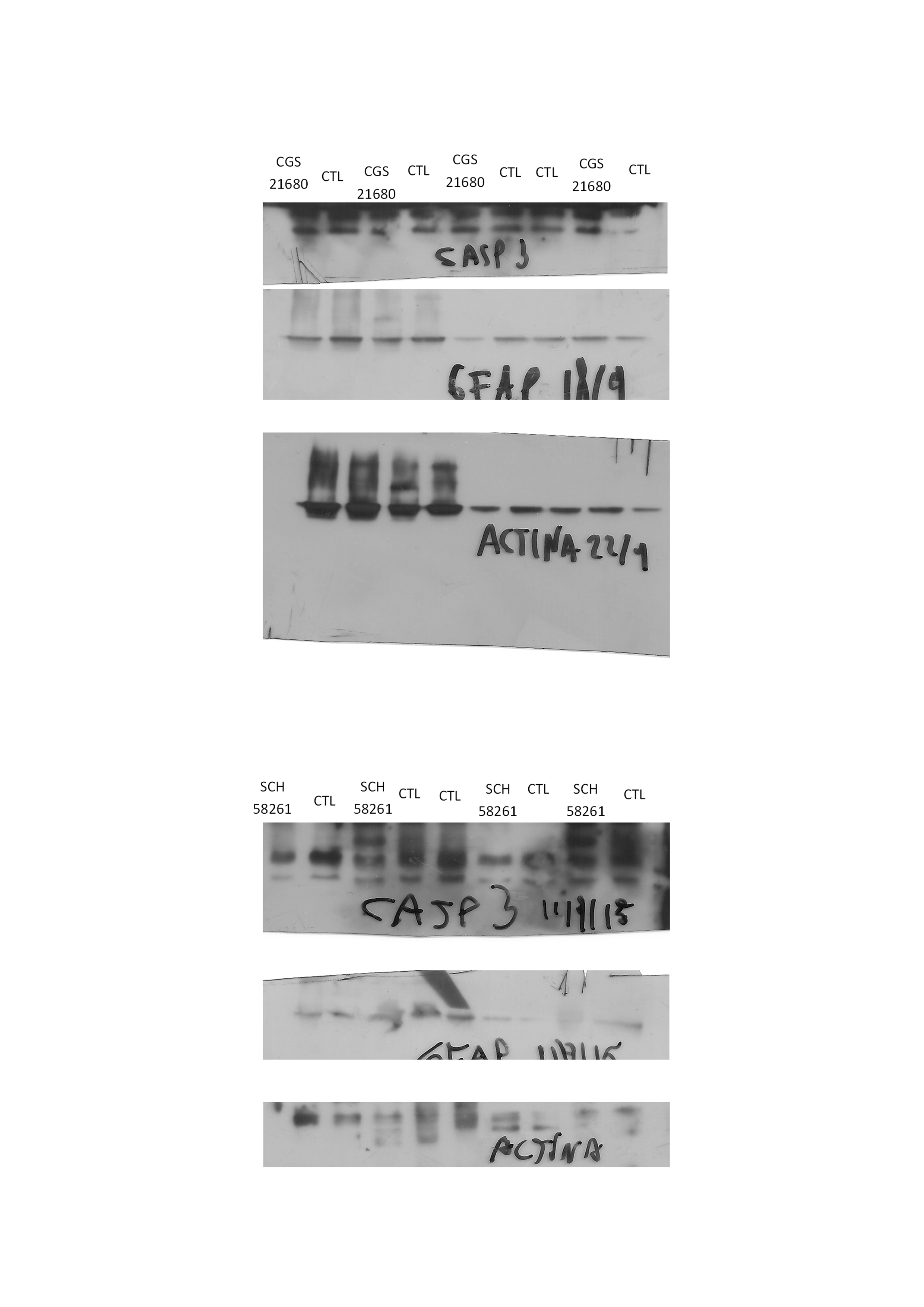

Supplement: Supplementary file 2 [file Image4.TIF]

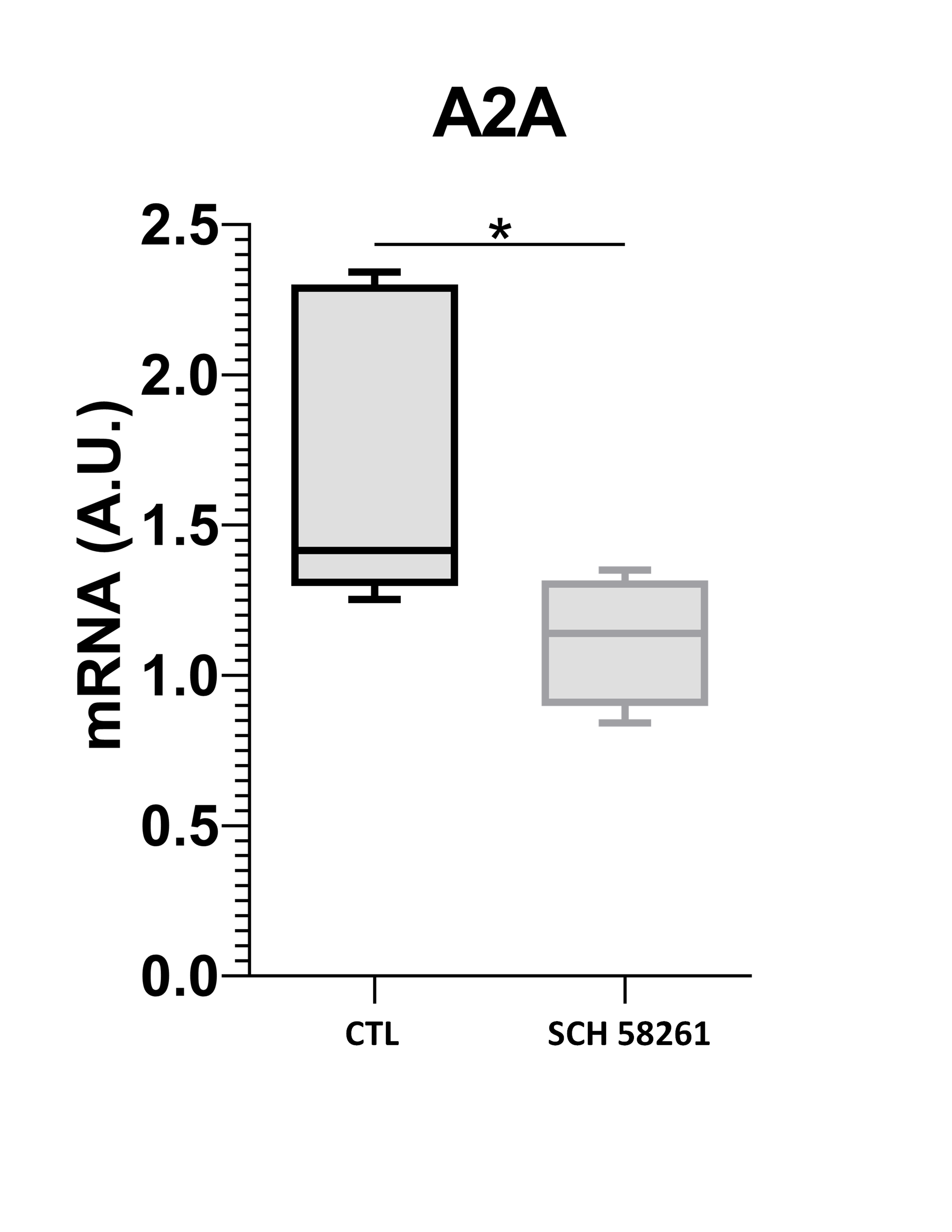

Supplement: Supplementary file 3 [file Image2.TIF]

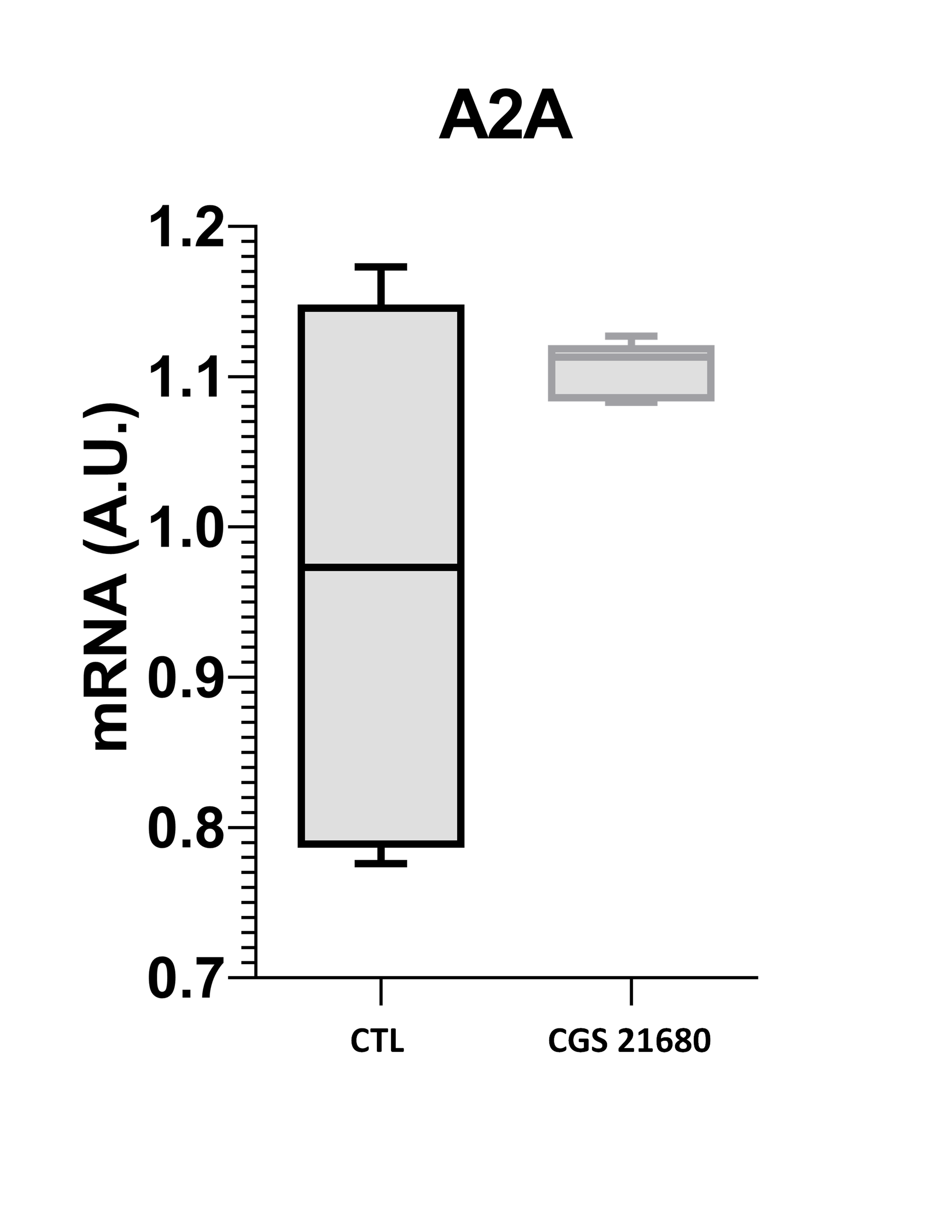

Supplement: Supplementary file 4 [file Image1.TIF]
